# Short-term preliminary PROs results from HIPOCRAT-ESK study: a cohort, non-interventional, prospective, real-world study of functional remission in TRD patients treated with Esketamina nasal spray

**DOI:** 10.1192/j.eurpsy.2026.11419

**Published:** 2026-07-31

**Authors:** T. Caraballo Lopez, M. García Dorado, N. Cardoner Álvarez, Á. Ibáñez Cuadrado, J. Vendrell Serres, R. Gómez-Juanes

**Affiliations:** 1 Neuroscience; 2 Johnson & Johnson, Madrid; 3 Hospital Sant Pau i Santa Creu, Barcelona; 4 Hospital Ramón y Cajal, Madrid; 5 Hospital Vall d’Hebrón, Barcelona; 6 Hospital Son Espases, Palma de Mallorca, Spain

## Abstract

**Introduction:**

Treatment-resistant depression (TRD), defined as failure to achieve remission after two or more antidepressant treatments, affects an estimated 55% of individuals with major depression disorder (MDD). TRD patients experience a higher burden of illness, including diminished cognitive functioning, decreased workplace performance, increased suicide risk, and higher likelihood of developing comorbidities. Furthermore, TRD is associated with an elevated socioeconomic burden due to increased healthcare resource utilization (HRU) and the need for more intensive treatments.

**Objectives:**

To report the initial PROs results of HIPOCRAT-ESK, the first real-world, prospective study evaluating functional remission of TRD patients treated with ESK NS based in Spanish clinical practice

**Methods:**

HIPOCRAT-ESK is a cohort, non-interventional, prospective, real-world study evaluating functions remission as a primary endpoint in TRD patients treated with ESK NS as per the Spanish clinical practice including 300 TRD patients with a prospective 12-months follow up where the patient’s perspective about clinial and quaility of life impact is colleted with PHQ-9 and EQ-5D-5L questionnaires. We present the short-term results of these two PROs over the first months of treatment of the first 25% of total sample recruited.

**Results:**

83 patients were included in the ITT analysis. Baseline characteristics show a severely impacted (both at clinical and QoL level) sample as noted by the basal mean PHQ-9 score (20.64) and EQ-5D-5L (36.45). Despite the worsened prognosis of TRD population, patients treated with ESK NS as per clinicla practice, reported positive evolution soon after treatment initiation. The percentage of patients auto-classified as severely depressed in the beginning decreased from 63.83% to 32.86% by day 28 (end of ESK NS induction; Table 1). In parallel, no/minimal depressed and mildly depressed categories increased from 0% to 4.29% and from 1.20% up to 14.29%, respectively. QoL results (reported by EQ-5D-5L), showed a marked positive impact in QoL after ESK NS treatment initiation, with increasing number of patients reporting no problems/slight problems in every of the 5 questionnaire’s dimension during the induction phase.

**Table 1.** PHQ-9 total score evolution Table 1. Long description.

ITT population (%)			
Induction phase day	Day 0	Day 15	Day 28
No/minimal depression	0,00	2,60	4,29
Mild depression	1,20	9,09	14,29
Moderate depression	6,02	31,17	22,86
Moderately severe depression	28,92	19,48	25,71
Severe depression	63,86	37,66	32,86

**Conclusions:**

Preliminary PROs short-term analysis shows promising efficacy results, with auto-perceived improvements reported as soon as second administration session, highlighting the importance of and early-driven clinical improvement and the key role of patient`s voice in clinical research

**Disclosure of Interest:**

T. Caraballo Lopez: None Declared, M. García Dorado Employee of: Johnson & Johnson Full-time employee, N. Cardoner Álvarez Grant / Research support from: Has recieved Research support from Johnson & Johnson, Consultant of: Has participated as consultant for Johnson & Johnson, Á. Ibáñez Cuadrado Grant / Research support from: Has recieved research support from Johnson & Johnson, Consultant of: Has participated as consultant fro Johnson & Johnson, J. Vendrell Serres Grant / Research support from: Has recieved research support from Johnson & Johnson, Consultant of: Has participated as consultant for Johnson & Johnson, R. Gómez-Juanes Grant / Research support from: Has recieved research support from Johnson & Johnson, Consultant of: Has participated as consultant for Johnson & Johnson

## Long descriptions

### Table 1. Long description

The table tracks the evolution of P H Q 9 total scores for an I T T population across Day 0, Day 15, and Day 28.

* No or minimal depression: 0.00 percent at Day 0, increasing to 2.60 percent at Day 15 and 4.29 percent at Day 28.

* Mild depression: 1.20 percent at Day 0, increasing to 9.09 percent at Day 15 and 14.29 percent at Day 28.

* Moderate depression: 6.02 percent at Day 0, peaking at 31.17 percent at Day 15, then decreasing to 22.86 percent at Day 28.

* Moderately severe depression: 28.92 percent at Day 0, decreasing to 19.48 percent at Day 15, then increasing to 25.71 percent at Day 28.

* Severe depression: 63.86 percent at Day 0, decreasing significantly to 37.66 percent at Day 15 and further to 32.86 percent at Day 28.

Navigate back to Table 1..

